# Elevated Plasma Levels of NET Components in Men with Severe COVID‐19 Correlates to Increased Amounts of IL‐18

**DOI:** 10.1002/eji.202451546

**Published:** 2025-05-09

**Authors:** Emelie Backman, Remigius Gröning, Alicia Lind, Christoffer Granvik, Hinnerk Eilers, Anna Lange, Clas Ahlm, Sara Cajander, Mattias N. E. Forsell, Johan Normark, Constantin F. Urban

**Affiliations:** ^1^ Department of Clinical Microbiology Umeå University Umeå Sweden; ^2^ Umeå Centre for Microbial Research (UCMR) Umeå University Umeå Sweden; ^3^ Department of Diagnostics and Intervention Umeå University Umeå Sweden; ^4^ Laboratory Medicine Region Västerbotten Norrland University Hospital Umeå Sweden; ^5^ Department of Infectious Diseases Faculty of Medicine and Health Örebro University Örebro Sweden

**Keywords:** COVID‐19, disease severity, IL‐18, neutrophil extracellular traps, sex‐dependent

## Abstract

Severe COVID‐19 disease is accompanied by high plasma levels of proinflammatory, prothrombotic NETs, and NET‐inducing cytokine IL‐18. We found that both, IL‐18 and NETs, are elevated in men with severe disease, but not in women of the same category. Our findings warrant further investigation of sex‐related differences in SARS‐CoV‐2 infection.

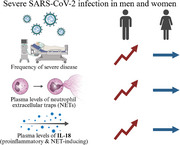

Severe complications of coronavirus disease 2019 (COVID‐19) are caused by dysregulated immune responses and characterized by hyperinflammatory states with thrombotic events [1]. Neutrophil extracellular traps (NETs), web‐like structures made of chromatin and decorated with antimicrobial proteins, like myeloperoxidase (MPO), are released to ensnare and restrict microbial pathogens [2]. In hyperinflammatory states, NETs promote immunothrombosis [3] and increase mortality [4]. Here, we investigated plasma from COVID‐19 patients for MPO‐DNA complexes (MDCs) indicative of NETs and proinflammatory cytokines with a focus on sex‐based differences.

We analyzed plasma from 206 participants, of which 86 (42%) were women, at the University Hospitals of Umeå and Örebro with PCR‐confirmed, severe acute respiratory syndrome coronavirus 2 (SARS‐CoV‐2) infection (Table S1–S3). Additionally, plasma from 77 anonymous healthy controls was included. MDC quantification in plasma by sandwich ELISA revealed higher levels in patients compared with healthy controls (Figure 1A) confirming previous findings [5]. Principal component analyses (PCA) comparing 39 cytokine and MDC levels in 280 plasma samples from 91 patients revealed differential clustering of patients with mild and severe disease (Figure 1B) up to 30 days after onset (Figure S2) for both sexes (Figure S3). Notably, levels of MDCs correlate with pro‐ and anti‐inflammatory cytokines IL‐6, TNFSF12, TNFSF10, and LT‐α, but not with case severity (Figure S4).

**FIGURE 1:**
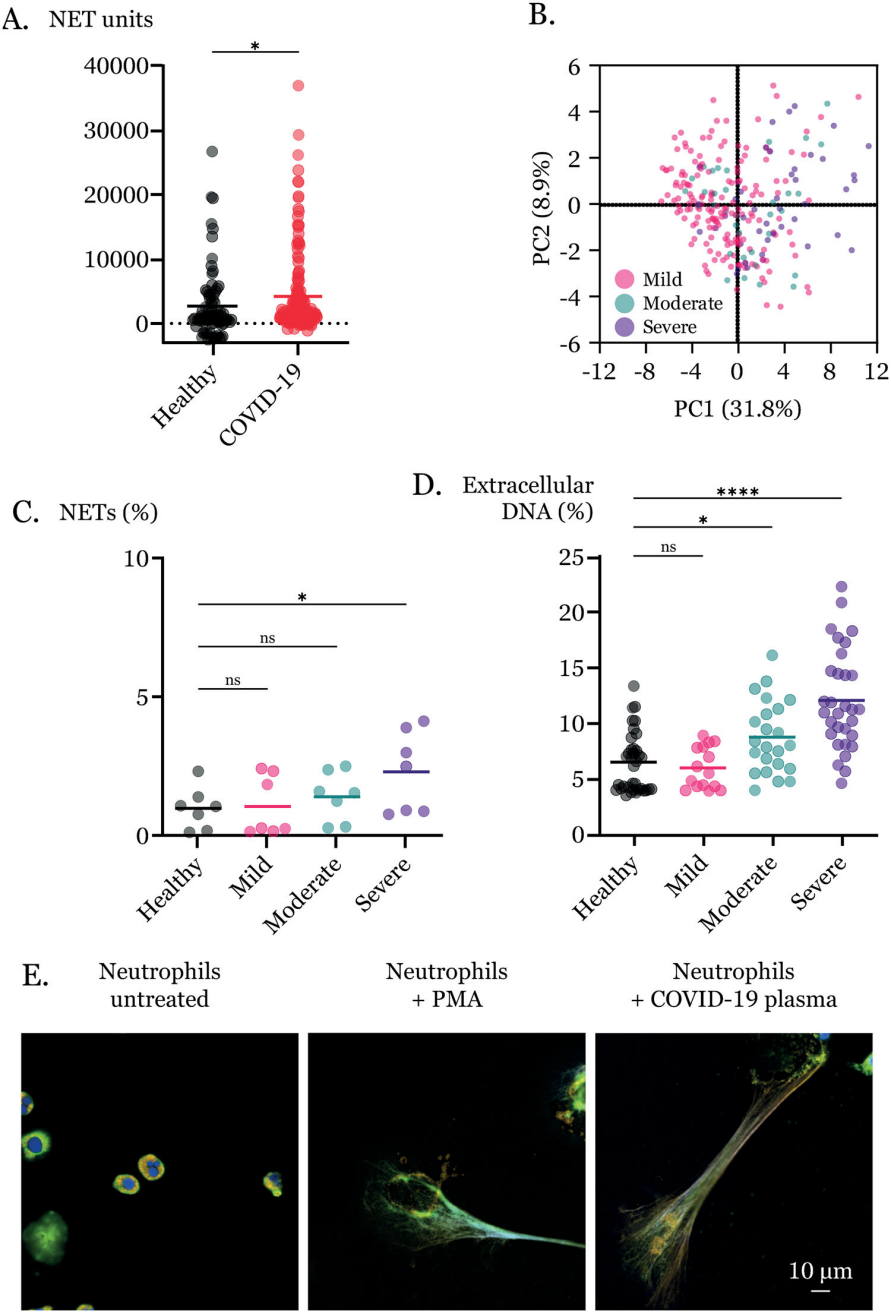
(A) MDCs in plasma quantified by sandwich ELISA. Maximum values within 30 days after onset for patients (*n* = 190) and healthy controls (*n* = 77). (B) PCA of cytokines in 280 plasma samples within 8 months after onset. Peripheral blood levels of 39 cytokines and MDCs. Loadings and proportion of variance in Figure S1. (C) Induced NETs after treatment with plasma (5 h) quantified microscopically by nucleus size. Mean values of neutrophil donors (*n* = 7) treated with patient or healthy control plasma. (D) Induced NETs quantified by extracellular DNA as percentage of positive control. Mean values of five donors treated with plasma from patients with mild (*n* = 8), moderate (*n* = 13), severe (*n* = 7) disease, or healthy controls (*n *= 29). (E) Representative micrographs with staining for DNA (DAPI), MPO (Alexa546), and neutrophil elastase (Alexa488), and images of separate stains are shown in Figure S5. Scale bars represent 10 µm. Plasma samples for (C), (D), and (E) were collected within 90 days after onset. Statistical significance was determined by (A) Mann–Whitney test, (C) Friedman test with Dunn's multiple comparisons, and (D) Kruskal–Wallis test with Dunn's multiple comparisons (*p ≤ 0.05, ****p ≤ 0.0001).

To determine whether plasma from COVID‐19 patients contains NET‐inducing factors, we treated neutrophils with plasma for 5 h, fixed and stained the cells for microscopy‐based NET quantification. We found that COVID‐19 plasma triggered NET release above healthy controls (Figure S5) and incremental with disease severity (Figure 1C). An independent assay, using a fluorescent, cell‐impermeable DNA dye, confirmed COVID‐19 plasma NET induction and severity‐dependent increases (Figure 1D). Morphologies of COVID‐19‐induced NETs resemble those triggered by NET inducer phorbol 12‐myristate 13‐acetate (Figure 1E).

To investigate whether COVID‐19‐induced NETs use pathways established to be involved in the NET formation, we used reactive oxygen species (ROS) scavenger tempol, Akt inhibitor XI (Figure 2A), and Syk inhibitor piceatannol (Figure S6). Each treatment reduced NET formation indicating that ROS and signaling through Akt and Syk contributed to COVID‐19‐induced NET formation and suggesting that pharmacological targeting of NETs is possible.

**FIGURE 2:**
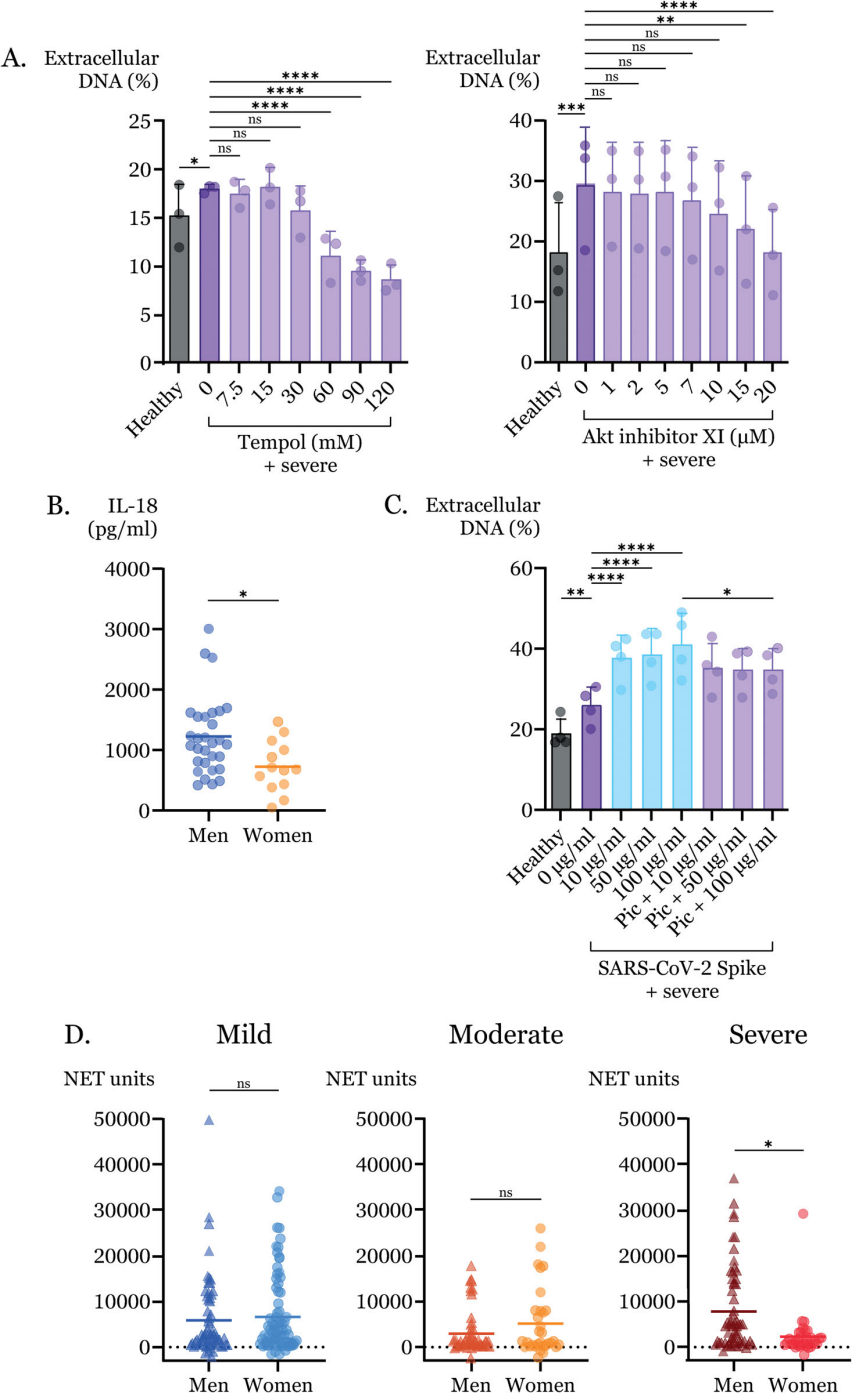
(A) Neutrophils from healthy donors treated with inhibitor (15 min) followed by plasma (5 h). Induced NETs quantified by extracellular DNA as percentage of positive control. Mean values of neutrophil donors (*n* = 3) each treated with plasma from six patients with severe COVID‐19 or four healthy controls. (B) IL‐18 concentration quantified by ELISA. Maximum values within 30 days after onset for men (*n* = 30) and women (*n* = 13) with severe disease. (C) Induction of NETs as in Figure 2A. Mean values of donors (*n* = 4) each treated with plasma from three patients with severe COVID‐19 or 1 healthy control in the presence of spike protein with or without Piceatannol (Pic), a Syk inhibitor, (10 µM). Controls in Figure S8. (D) MDCs in plasma quantified by sandwich ELISA. Mean value matched at each sample day, within 120 days after onset from patients with mild (*n* = 110), moderate (*n* = 34), or severe disease (*n* = 48). Statistical significance was determined by (A) two‐way ANOVA with Dunnett's multiple comparisons test, (B) Mann–Whitney test, (C) repeated measures one‐way ANOVA with Šídák's multiple comparisons test, and (D) mixed‐effects model with Šídák's multiple comparisons test (*p ≤ 0.05, **p ≤ 0.01, ***p ≤ 0.001, ****p ≤ 0.0001).

Although overall case numbers of diagnosed COVID‐19 were similar for men and women, men were three times more likely to require intensive care [6]. In Sweden, twice as many men received COVID‐19‐related intensive care (The Swedish Intensive Care Registry). We examined differences in cytokine levels in plasma within the first 30 days. Confirming prior results, several previously reported proinflammatory cytokines [7, 8] were elevated in severe cases (Figure S7), among those IL‐10, IL‐6, CCL7, and IL‐18 were higher in men than in women. However, only IL‐10 reached the significance threshold. We focused on IL‐10 and IL‐18, cytokines with direct effects on neutrophil activation. While IL‐10 levels were below the detection limit, IL‐18, known to induce NET formation [9], showed significantly higher levels in men compared with women in a separate assay (Figure 2B).

In agreement with elevated IL‐18, MDC levels showed sex‐dependent differences (Figure S9). A detailed comparison of consecutive samples revealed significantly increased MDC levels in the plasma of men with severe disease (Figure 2D) with elevation throughout disease progression (Figure S10). No increases were found in plasma from women with severe disease or in patients with mild and moderate disease.

Antibodies form immune complexes with SARS‐CoV‐2 proteins [10], triggering NET formation via Fc receptors on neutrophils and SARS‐CoV‐2 spike protein can induce IL‐18 expression [8]. We hypothesized that spike protein contributes to COVID‐19‐induced NET formation in severe cases. We added recombinant protein to plasma‐mediated NET induction assays and showed that NET formation increased upon addition of spike protein (Figure 2C). Blockage of Syk abrogated the spike‐boosted NET formation suggesting that the effect was due to Fc receptors.

Our study corroborates a correlation of NETs with severe disease and supports the use of NETs as a potential clinical marker and therapeutic target for severe COVID‐19 in a sex‐dependent and IL‐18‐related manner. The number of samples from severe disease patients sets limits, and we advise investigating sex‐dependent NET formation in larger cohorts.

## Conflicts of Interest

The authors declare no conflicts of interest.

### Peer Review

The peer review history for this article is available at https://publons.com/publon/10.1002/eji.202451546.

## Supporting information



Supporting Information

## Data Availability

The CoVUm data are not publicly available, according to Swedish data protection laws and the terms of ethical approval stipulated by the Ethical Review Authority of Sweden. Access to this data is organized according to procedures complying with Swedish law and proposals must be submitted to the corresponding authors. After evaluation, data access is contingent on vetting by the Ethical Review Authority of Sweden, according to the Act (2003:460) concerning the Ethical Review of Research Involving Humans.
