# Supplementary material for: Elevated Plasma Levels of NET Components in Men with Severe COVID‐19 Correlates to Increased Amounts of IL‐18
Source: Eur J Immunol. 2025 May 9;55(5):e202451546. doi: 10.1002/eji.202451546 (PMC12064869; doi:10.1002/eji.202451546)
Supplement: Supplementary file 1 — Supporting Information [file EJI-55-e202451546-s001.pdf]

Supporting Information to

Elevated plasma levels of NET components in men with severe COVID-19 correlates to increased amounts of IL-18

Emelie Backman[1,2], Remigius Gröning[1,2], Alicia Lind[2,3], Christoffer Granvik[1], Hinnerk Eilers[4], Anna Lange[5], Clas Ahlm[1,2], Sara Cajander[5], Mattias N. E. Forsell[1,2], Johan Normark[1*], Constantin F. Urban[1,2*]

[1]Department of Clinical Microbiology, Umeå University, Umeå, Sweden

[2]Umeå Centre for Microbial Research (UCMR), Umeå University, Umeå, Sweden

[3]Department of Diagnostics and Intervention, Umeå University, Umeå, Sweden

[4]Laboratory Medicine, Region Västerbotten, Norrland University Hospital, Umeå, Sweden

[5]Department of Infectious Diseases, Faculty of Medicine and Health, Örebro University, Örebro, Sweden.

[*]Equally contributing senior authors

[#]Corresponding author: Constantin F. Urban, constantin.urban@umu.se

Keywords: COVID-19, Neutrophil extracellular traps, IL-18, disease severity, sex-dependent

Abbreviations

COVID-19: Coronavirus disease 2019

DNA: Deoxyribonucleic acid

ELISA: Enzyme-linked immunosorbent assay

MDCs: MPO-DNA complexes

MPO: Myeloperoxidase

NETs: Neutrophil extracellular traps

PCA: Principal component analysis

Materials and methods

Study design and cohort

Data and human biological material were retrieved from a prospective multi-centre cohort study (CoVUm, ClinicalTrials.gov ID NCT04368013). Patients were prospectively enrolled between April 2020 and June 2021. All patients had a confirmed COVID-19 diagnosis based on PCR testing. Hospitalized (≥ 18 years of age) and non-hospitalized (≥ 15 years) patients with a positive PCR test for SARS-CoV-2 were eligible for enrollment. Hospitalized patients due to acute COVID-19 infection were enrolled at the Departments of Infectious Diseases and the intensive care units (ICU) at Örebro University Hospital and University Hospital of Umeå. Non-hospitalized patients that fulfilled the inclusion criteria were prospectively enrolled at the infectious diseases' outpatient clinic at University Hospital of Umeå. The patients were followed up over time and blood samples were collected at submission, 2-, 4-, 8 weeks, 3-, 6 months and 1-year after disease onset. Samples from patients at inpatient care were collected every other day. Infection severity was classified depending on level of supplemental oxygen therapy in accordance with WHO clinical progression scale [1].

Healthy individuals were recruited through the local blood bank (Umeå, Region Västerbotten). The requirement for an ethical permit was waived since all samples were, upon fully informed consent anonymized and could not be traced back to the donors.

Blood samples were collected into EDTA tubes and plasma was separated by centrifugation at 1600 g for 10 minutes at 4°C. Samples were divided into small aliquots and stored at -80°C until the time of testing.

Neutrophil isolation from whole blood

Polymorphonuclear leukocytes (PMNs) or neutrophils were isolated from venous blood according to [2]. Briefly, neutrophils were separated from blood with two gradient centrifugations. The first one uses Histopaque 1119 (Sigma-Aldrich, 11191) to remove red blood cells while the second one uses a discontinuous gradient of Percoll (Sigma-Aldrich, GE17-0891-01) that separate neutrophils from monocytes, lymphocytes, and residual red blood cells. To ensure that all red blood cells were removed, cells were treated with RBC lysis buffer (BioLegend, 420301) for 1 minute. After each centrifugation, cells were washed with Phosphate buffered saline (PBS) supplemented with 0.5% Human serum albumin (HSA) (CSL Behring). Purified neutrophils were resuspended at desired concentrations in RPMI-1640 medium (Lonza, 12-918F) supplemented with 10 mM HEPES (Here after called RPMI).

Standard for NET units

Serving as a standard, NET units are derived by quantification of NETs from a defined number of neutrophils stimulated with PMA [3].

A Flat-Bottom 96-Well Plate (Thermo Scientific, 3355) was coated with 0.001% Poly-L-lysine (Sigma-Aldrich, P8920) for 2 minutes and then washed 2 times with 100 µl MilliQ water before 50,000 purified neutrophils were seeded into each well. Neutrophils were treated with 100 nM PMA (Sigma-Aldrich P8139) to induce NET formation and plate was incubated for 5 hours at 37°C with 5% CO_2.

All wells were treated with DNase I (Sigma-Aldrich, DN25) (3.94 U/ml) and incubated for 15 minutes in 37°C before the addition of 10 mM EDTA to stop the reaction. The plate was centrifuged for 10 minutes at 200 g, supernatants were transferred to Eppendorf tubes and centrifuged again for 5 minutes

at 300 g before the final collection of supernatants. NET standards were aliquoted and frozen to -80°C until use in ELISA.

Quantification of MPO-DNA complexes

MPO-DNA complexes (MDCs) were quantified using several reagents from the Cell Death Detection ELISA^{PLUS} kit (Roche, 11920685001). A Flat-Bottom 96-Well Plate was coated with anti-human MPO antibody (R&D Systems, AF3667) (1 µg/ml) in Coating buffer (Sodium carbonate 0.05 M, pH 9.5-9.7) overnight at 4°C.

The plate was washed two times with Wash buffer (0.05% Tween 20 (VWR, 28829.296) in PBS) and blocked with Blocking buffer (1% Bovine serum albumin (Sigma-Aldrich, A7906) in Wash buffer) for 2 hours at room temperature.

The plate was washed 5 times before addition of 20 µl patient plasma (10%) and NET standard in a 1:2 dilution series in Blocking buffer. 80 µl Anti-DNA-POD (Cell Death kit) was added to all wells and plate was incubated for 2 hours at room temperature with 300 rpm shaking.

After 5 more washes the plate was developed with 100 µl 3,3′,5,5′-Tetramethylbenzidine (TMB) substrate (Sigma-Aldrich, T0440) followed by the same volume of Stop Reagent for TMB Substrate (Sigma-Aldrich, S5814). Absorbance was measured at wavelength 450 nm using a Varioskan Flash plate reader (Thermo Scientific). Data were normalized to in vitro–prepared NET standards included on every plate and quantified based on the number of NET forming cells (NET units).

Quantification of NET formation (immunofluorescent microscopy)

NET induction

Coverslips were added to a 24-well plate and coated with Poly-L-lysine (0.001%) for 2 minutes and then washed two times with MilliQ water and one time with RPMI medium. 100,000 isolated neutrophils were seeded into each well and incubated for 30 minutes in room temperature before addition of stimulus: RPMI (unstimulated), PMA (Sigma-Aldrich, P8239) (100 nM) and plasma (10%). Plates were incubated for 0 and 5 hours at 37°C with 5% CO_2. Cells were fixated overnight in 4°C by addition of paraformaldehyde (PFA) (2%).

Staining

Coverslips were washed 3 times with Wash buffer (0.05% Tween 20 in PBS), permeabilized with Triton (0.5%) for 1 minute and washed 3 more times for 5 minutes. Coverslips were blocked with Blocking buffer for 30 minutes in a humidity chamber and then incubated with primary antibodies (Anti-Neutrophil Elastase (Mouse) (Dako, M0752) and Anti-MPO (Rabbit) (Dako, A0398) (1:200)) for 1 hour at 37°C in a humidity chamber. Coverslips were washed 3 times before incubation with secondary antibodies (Goat anti-Mouse IgG (H+L) Cross-Adsorbed Secondary Antibody, Alexa Fluor™ 488 (Invitrogen, A-11001) and Goat anti-Rabbit IgG (H+L) Highly Cross-Adsorbed Secondary Antibody, Alexa Fluor™ 546 (Invitrogen, A-11035)) (10 µg/ml) for 1 hour at 37°C in a humidity chamber in the dark. Coverslips were washed 3 times for 5 minutes followed by staining with DAPI (1 µg/ml) for 5 minutes at room temperature and then washed 5 times for 5 minutes. Coverslips were mounted on glass slides using Prolong Diamond Antifade Mountant (Invitrogen, P36961) or Fluorescence Mounting Medium (Dako, S3023).

Imaging and analysis

Slides were photographed with a Cytation 5 Cell Imaging Multi-Mode Reader (BioTek) at 20X magnification as 4×4 tiles with a minimum of 700 cells per condition. ImageJ [4] was used to quantify

percentage of NET forming cells in respect to area of each cell nuclei where manual adjustment of a set threshold was applied to each condition. For each condition, neutrophils from one donor were treated with plasma from one COVID-19 patient or healthy control. Percentage of NET formation was averaged for each patient group (mild, moderate or severe) and matched by neutrophil donor in the analysis to account for donor variations.

Micrographs were photographed with a Nikon 91i Eclipse microscope with C1 Plus confocal using NIS-E AR and EZ-C1 software at 60X magnification.

SARS-CoV-2 spike protein production

The plasmid encoding 2019-nCoV S protein was provided by Jason McLellan and the details of this construct have been previously described [5].

The protein was produced using the Gibco ExpiCHO Expression System (Thermo Scientific). ExpiCHO cells were transfected with the plasmid using ExpiFectamine reagent and OptiPRO SF medium in a 6E6 cells/ml culture according to the manufacturer's protocol and subsequently grown for 8 days at 37°C with 8% CO_2 in 80% humidity at 100 rpm. ExpiCHO Enhancer with ExpiCHO Feed was added one day after transfection according to the manufacturer's protocol. Eight days after transfection, the supernatant was cleared of cells and debris by centrifugation at 1500 g at 4°C for 20 minutes and subsequent passage through a 0.2 µM filter. The filtered supernatant was adjusted to pH 7.4, mixed with 3 ml of His-pure Ni-NTA resin (Thermo Scientific) in a 1 l Nalgene Single-Use PETG Erlenmeyer Flask with Baffled Bottom (Thermo Scientific), and incubated at 4°C with 85 rpm overnight. The resin with the attached protein was collected in an Econo-Column Chromatography 2.5 × 20 cm column (Bio-Rad). The resin was washed with 50 ml imidazole/PBS pH 7.4 (20 mM) and then eluted with 30 ml of imidazole/PBS pH 7.4 (250 mM). The resulting eluate was concentrated, and the buffer exchanged to 50 mM TRIS, 250 mM NaCl pH 8.0 using Amicon Ultra-15 Centrifugal Filters (100 kDa cut-off). Protein purity was determined by SDS-PAGE and concentration was determined by a BCA Protein Assay kit (Thermo Scientific, 23227). Protein was stored at a concentration of 1 mg/ml at -80°C.

Quantification of NET formation (extracellular DNA)

Isolated neutrophils were seeded into a black 96-well plate at 50,000 cells per well. The cell impermeant dye SYTOX Green (Invitrogen, S7020) (0.5 µM) was added to each well followed by stimulus; plasma (10%), PMA (100 nM) or Triton X-100 (VWR, 28817.295) (1% in PBS). The plate was incubated in a FLUOstar Omega microplate reader (BMG LABTECH) for 5 hours at 37°C with 5% CO_2 and excitation/emission was measured at wavelengths of 504/523 nm.

For NET inhibition experiments, neutrophils were pre-treated with the following inhibitors: TEMPOL (Sigma-Aldrich, 176141), Akt Inhibitor XI (Cayman Chemical, 18604), BB-Cl-Amidine (Cayman Chemical, 17079), CLI-095 (InvivoGen, tlrl-cli95) or Piceatannol (Sigma-Aldrich, P0453-5MG) for 15 minutes in room temperature before addition of stimulus.

For NET induction with SARS-CoV-2 spike protein, plasma (10%) was mixed with different concentrations of spike protein or HSA (CSL Behring) (100 µg/ml) before addition. The mix was used to treat neutrophils from healthy controls for the duration of 5 hours.

Extracellular DNA was quantified by fluorescence intensity as percentage of Triton treated cells (100% control).

Cytokine analysis

Olink® sample preparation

Blood EDTA plasma samples drawn from COVID-19 patients were inactivated prior to shipping according to Olink® guidelines. 25 µl of each sample was inactivated for 2 hours at room temperature with Triton X-100 (1%). Samples were analyzed with the Olink® Target 48 service.

Olink® raw data preparation

Raw data resulting from the Olink® Target 48 measurement of patient blood plasma was obtained in the form of normalized protein expression (NPX). Cytokines containing significant amounts of missing data (about more than 25% of samples) were excluded from downstream analysis. Subsequently, a python script (found at https://github.com/RGroening/ olink_data_preparation_final/ blob/main/ 20230126_olink_data_preparation.py) was used within the Visual Studio Code development environment (version 1.85.1) to match samples to corresponding patient IDs, time after disease onset "Phase", demographic data, comorbidities, and treatments. The resulting DataFrame was saved as a CSV file and used for multivariate statistical analyses. In addition, another python script (https://github.com/RGroening/olink_data_preparation_final/blob/main/20230126_olink_data_preparation.py) was used to filter the maximum cytokine sample value for each cytokine, time period, and patient.

IL-18 and IL-10 cytokine sandwich ELISA

IL-18 and IL-10 levels were quantified in blood EDTA plasma samples drawn from COVID-19 patients with severe disease using the Human IL-18 ELISA Kit (ThermoFisher Scientific, BMS267-2) and Human IL-10 ELISA Kit (ThermoFisher Scientific, EHIL10) according to the manufacturer's guidelines using the supplied standards.

Statistical analysis of cytokine data

Statistical analysis of prepared Olink® and IL-18/IL-10 ELISA data was conducted in GraphPad Prism (version 10.3.1). Multivariate Olink® cytokine and matched clinical data was analyzed by principal component analysis and multiple Mann-Whitney tests with Holm-Šídák adjusted P values using otherwise program-standard parameters. Multivariate correlation analysis was performed using the Spearman rank correlation method.

Statistical analysis

All statistical analyses were conducted in GraphPad Prism (version 10.3.1). Trimmed mean was used as indicated in figure captions where the highest and lowest values were removed to ensure that the results were not influenced by single outliers.

Illustrative art work

We used BioRender.com and Adobe Illustrator to design the graphical abstract illustration.

Ethical considerations

The study was conducted in accordance with the Declaration of Helsinki. Written informed consent was obtained from all patients or, if unable to consent, next of kin. Ethical approval for the study was granted from Swedish Ethical Review Authority (approval number: 2020-01557).

Author contributions

Emelie Backman: Formal analysis, investigation, validation, visualization, writing – original draft; Remigius Gröning: Formal analysis, investigation, visualization, writing – review & editing; Alicia Lind: Data curation, project administration, resources, writing – review & editing; Christoffer Granvik: Data curation, formal analysis, methodology, validation, visualization, writing – review & editing; Hinnerk Eilers: Funding acquisition, project administration, resources; Anna Lange: Data curation, resources; Clas Ahlm: Funding acquisition, project administration, supervision, writing – review & editing; Sara Cajander: Project administration, resources, supervision; Mattias N. E. Forsell: Funding acquisition, project administration, resources, supervision, writing – review & editing; Johan Normark: Conceptualization, funding acquisition, methodology, project administration, resources, supervision, validation, writing – review & editing; Constantin F. Urban: Conceptualization, formal analysis, funding acquisition, methodology, project administration, resources, supervision, validation, writing – review & editing.

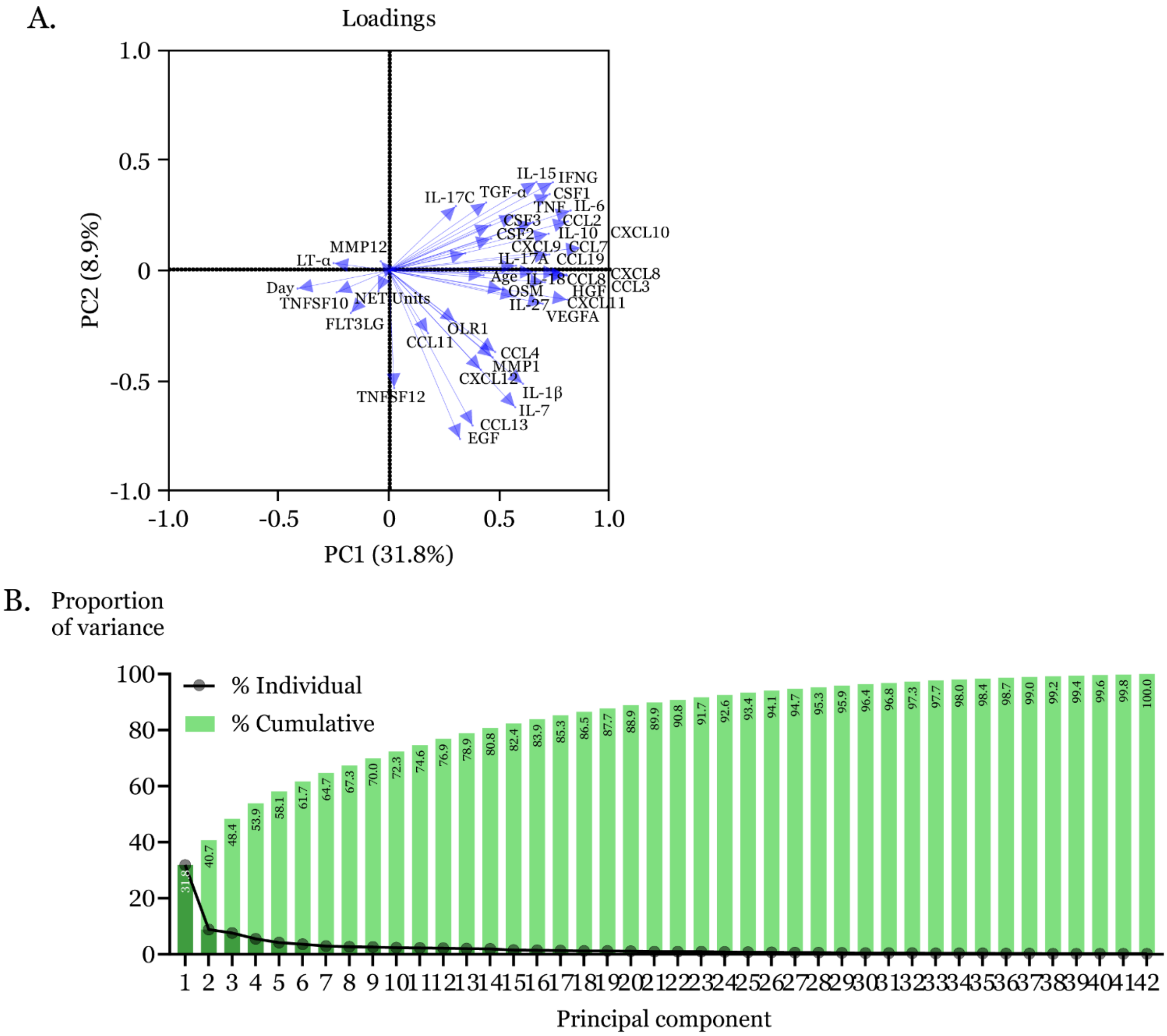

Figure S1

PCA of cytokines, MDCs, age and days after onset of COVID-19 patients. (A) Loadings and (B) proportion of variance of the first two principal components for Figure 1B performed on 280 plasma samples from 91 COVID-19 patients within 8 months after disease onset, analyzed for peripheral blood levels of 39 cytokines quantified by Olink® and MDCs quantified by sandwich ELISA.

1-10 days after disease onset

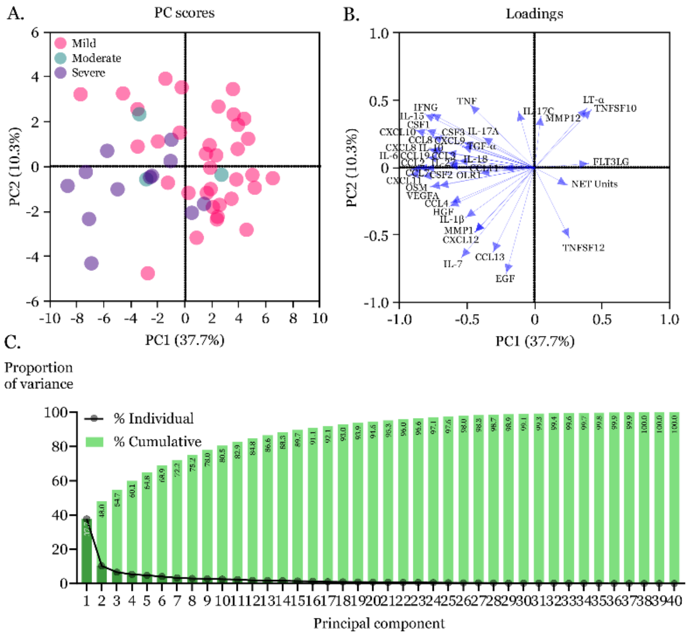

11-30 days after disease onset

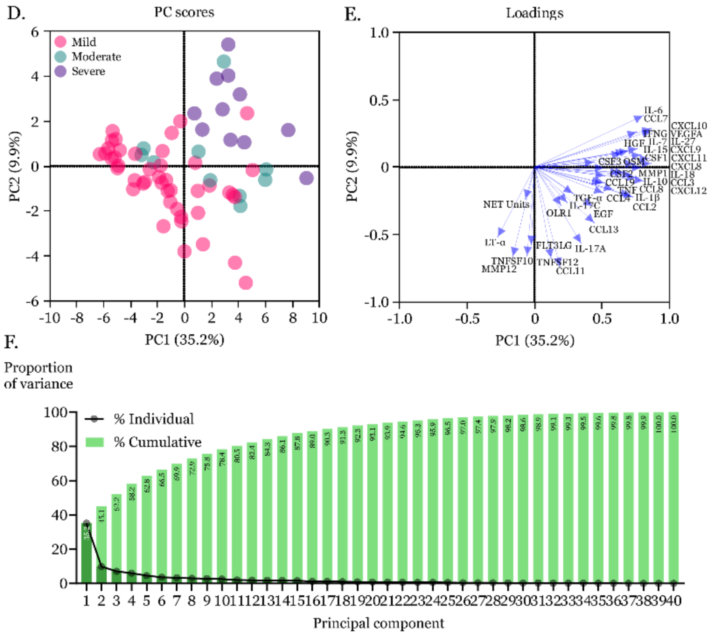

>30 days after disease onset

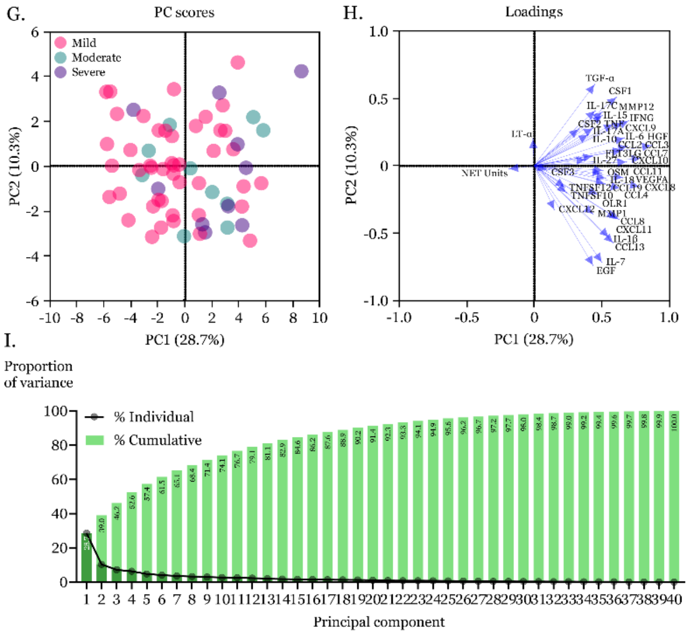

Figure S2

PCA of cytokines, MDCs, age and days after onset of COVID-19 patients. PC scores and loadings of the first two principal components and proportion of variance performed on samples obtained at (A, B, C) 1 to 10 days (n = 50), (D, E, F) 11 to 30 days (n = 65), and (G, H, I) 30 days to 8 months (n = 65) after disease onset. 280 plasma samples from 91 COVID-19 patients within 8 months after disease onset were analyzed for peripheral blood levels of 39 cytokines quantified by Olink® and MDCs quantified by sandwich ELISA.

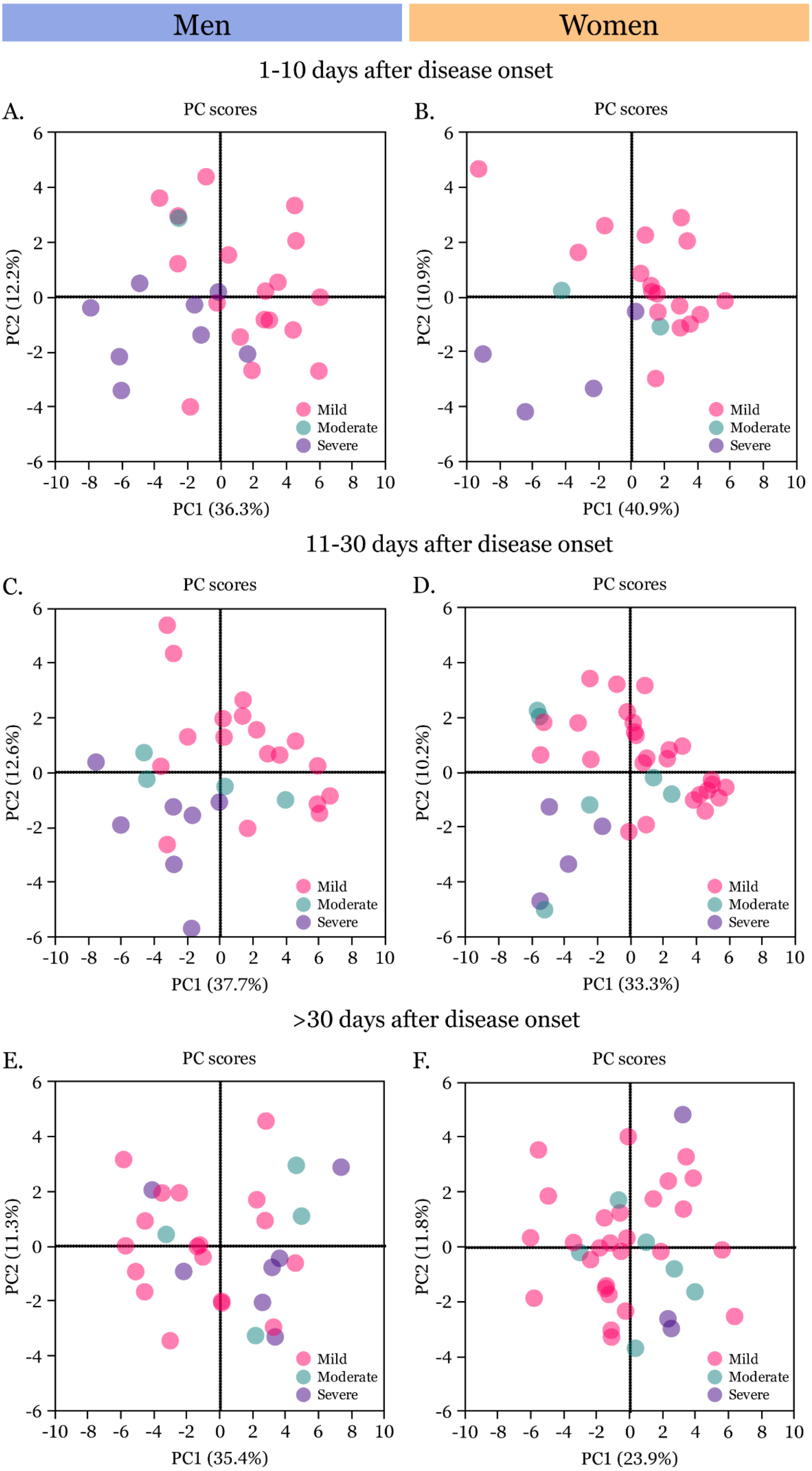
Men
Women
1-10 days after disease onset
A.
PC scores
PC2 (12.2%)
PC1 (36.3%)
B.
PC scores
PC2 (10.9%)
PC1 (40.9%)
11-30 days after disease onset
C.
PC scores
PC2 (12.6%)
PC1 (37.7%)
D.
PC scores
PC2 (10.2%)
PC1 (33.3%)
>30 days after disease onset
E.
PC scores
PC2 (11.3%)
PC1 (35.4%)
F.
PC scores
PC2 (11.8%)
PC1 (23.9%)
Mild
Moderate
Severe

Figure S3

PCA of cytokines, MDCs, age and days after disease onset of COVID-19 patients. PC scores of the first two principal components performed on samples obtained from men or women at (A, B) 1 to 10 days, (C, D) 11 to 30 days, and (E, F) 30 days to 8 months after disease onset. 280 plasma samples from 91 COVID-19 patients within 8 months after disease onset were analyzed for peripheral blood levels of 39 cytokines quantified by Olink® and MDCs quantified by sandwich ELISA.

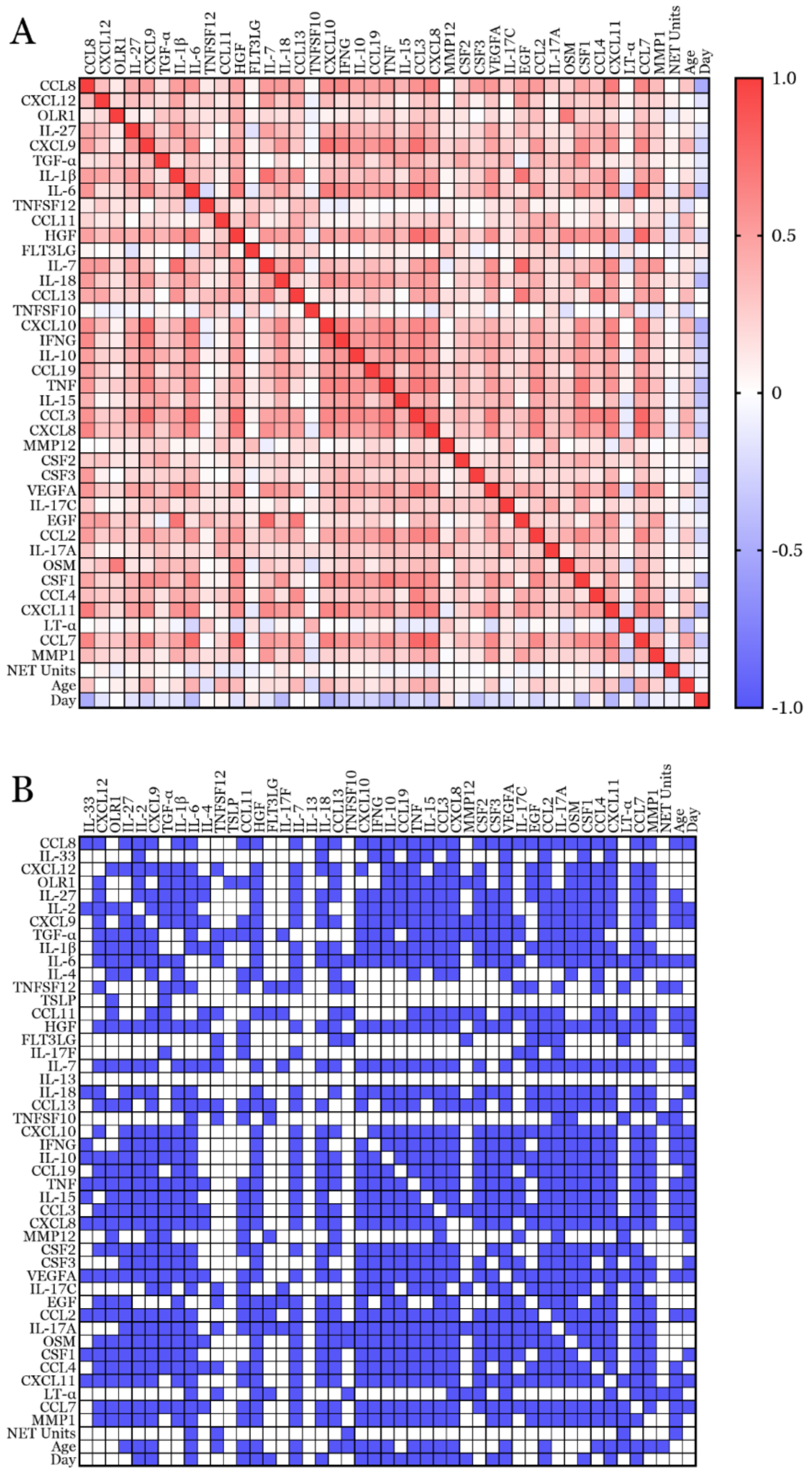

Figure S4

Correlation analysis of cytokines and MDCs, age and days after disease onset in COVID-19 patients within 30 days after disease onset. Cytokine levels were measured by Olink® and MDCs quantified by sandwich ELISA. Correlation matrix (A) displays the Spearman rank correlation coefficients between different factors in 151 samples. (B) P values of the corresponding Spearman correlation matrix. Matrix cells with a P value below 0.05 were depicted in blue.

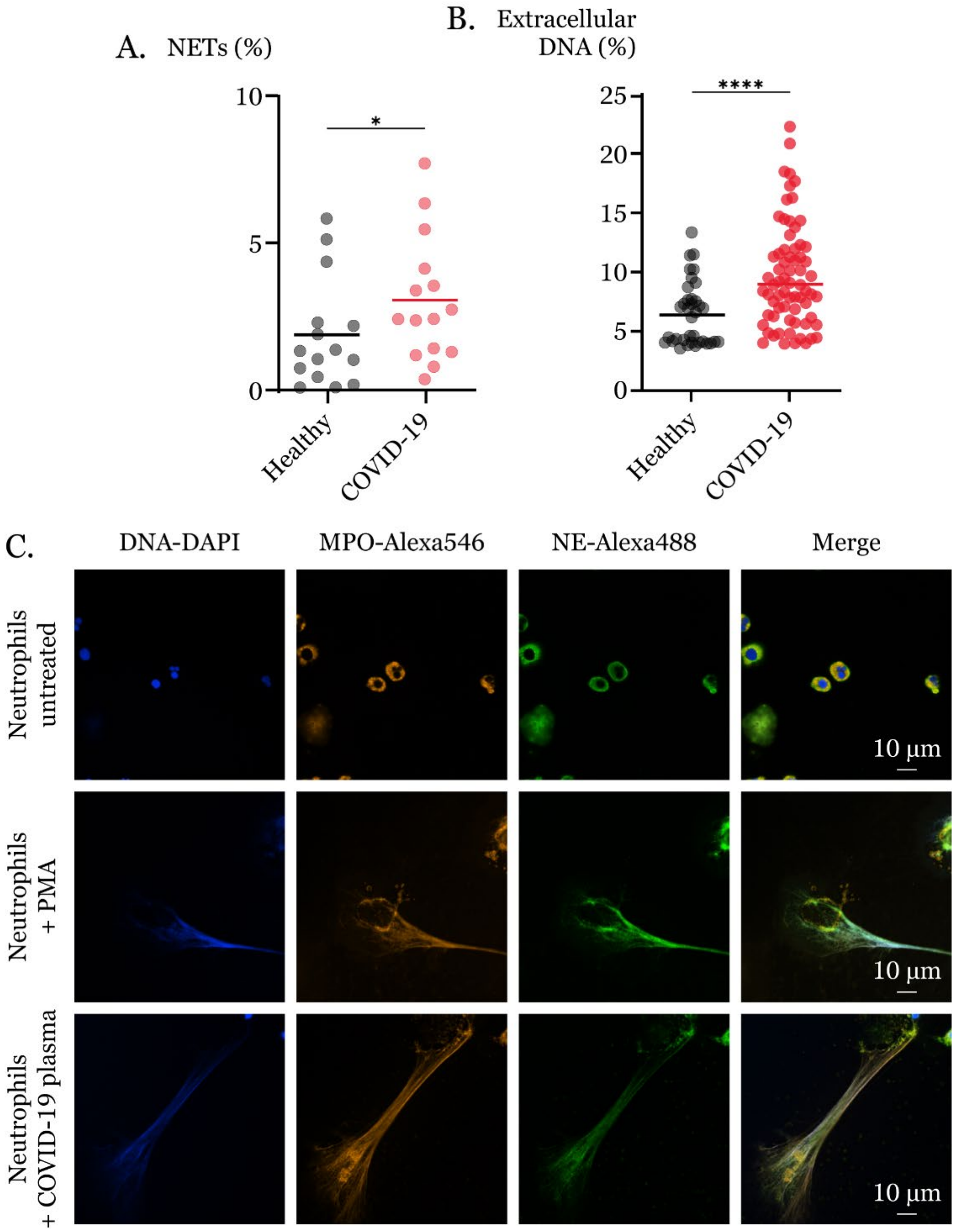

Figure S5

Neutrophils isolated from healthy donors treated with plasma (5 hours). Induced NET formation was determined by (A, C) image analysis of micrographs quantifying percentage of NET forming cells based on the nuclei size of each cell. (A) Mean values of neutrophil donors (n = 15) treated with plasma from COVID-19 patients or healthy controls. (B) NET formation quantified by released extracellular DNA as percentage of positive control. Mean value of 5 donors treated with plasma from patients (n = 28) or healthy controls (n = 29). (C) Representative micrographs of indirect immunofluorescence with staining for DNA (DAPI) and fluorescence immunostaining for MPO (Alexa546) and Neutrophil elastase (NE) (Alexa488). NETs were identified by colocalization of extracellular laminar DNA with MPO and NE. Scale bars represent 10 µm. Plasma samples were collected within 90 days after disease onset. Statistical significance was determined by (A) Paired t test and (B) Friedman test with Dunn's multiple comparisons test (*$p \leq 0.05$, ****$p \leq 0.0001$).

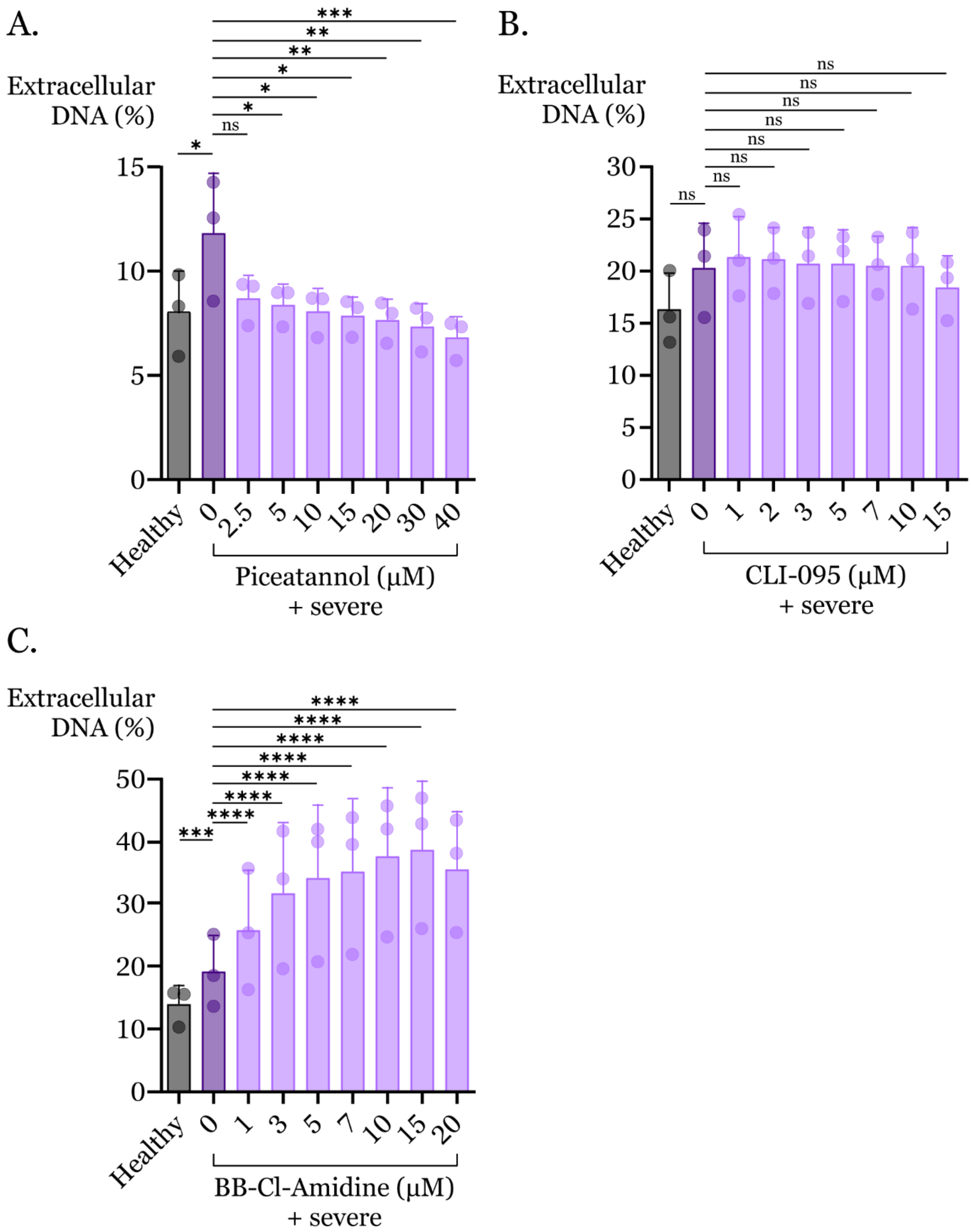

Figure S6

Neutrophils isolated from healthy donors treated with inhibitor (15 minutes) followed by plasma (5 hours). Induced NET formation was determined by released extracellular DNA quantified as percentage of positive control. Mean value of neutrophil donors (n = 3) each treated with plasma from 6 patients with severe COVID-19 or 4 healthy controls in the precence of (A) Piceatannol (Syk inhibitor), (B) CLI-095 (TLR4 inhibitor) and (C) BB-Cl-Amidine (PAD inhibitor). Statistical significance was determined by 2-way ANOVA with Dunnett's multiple comparisons test (*p ≤ 0.05, **p ≤ 0.01,***p ≤ 0.001, ****p ≤ 0.0001).

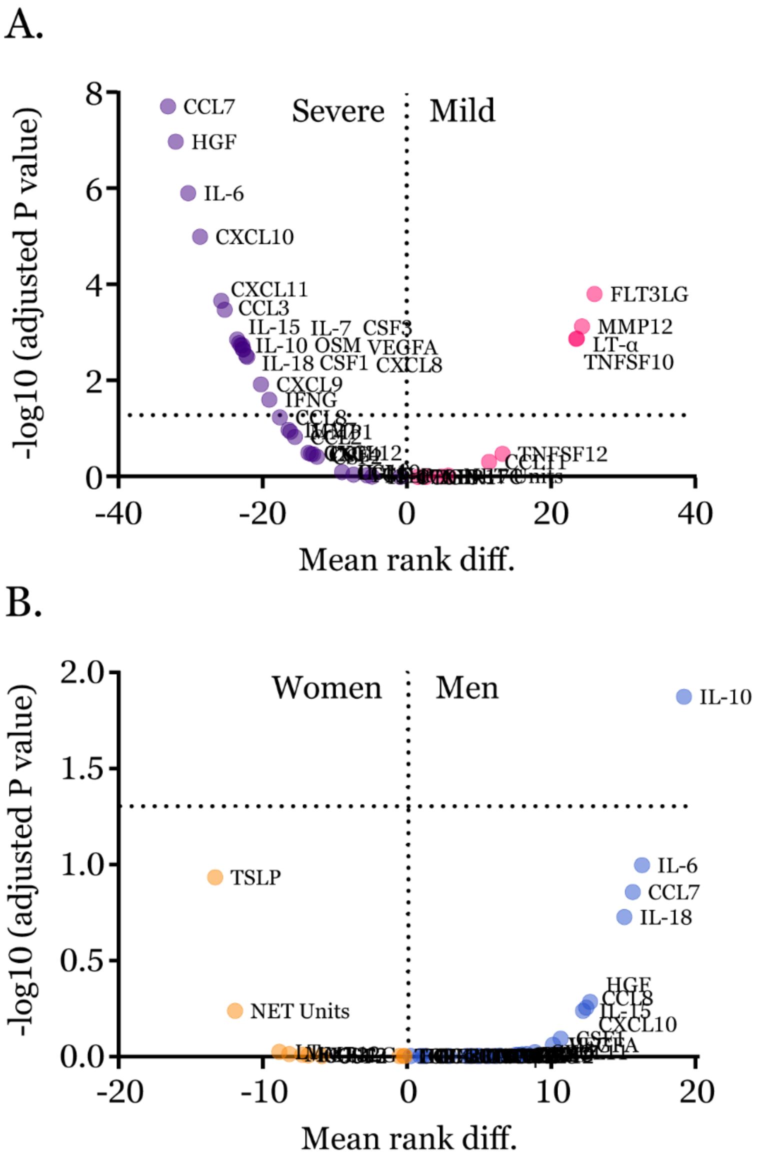

Figure S7

Differences in cytokine and MPO-DNA complexes (MDCs) in plasma from COVID-19 patients with (A) mild (n = 59) and severe (n = 19) disease or (B) men (n = 45) and women (n = 43) during the first 30 days after disease onset. 40 cytokines quantified by Olink® MDCs quantified by sandwich ELISA. Values from mean rank diff. and adjusted P value can be found in Table S4. Statistical significance was determined by Multiple Mann-Whitney tests with Holm-Šídák method. Horizontal, dashed line indicates p = 0.05.

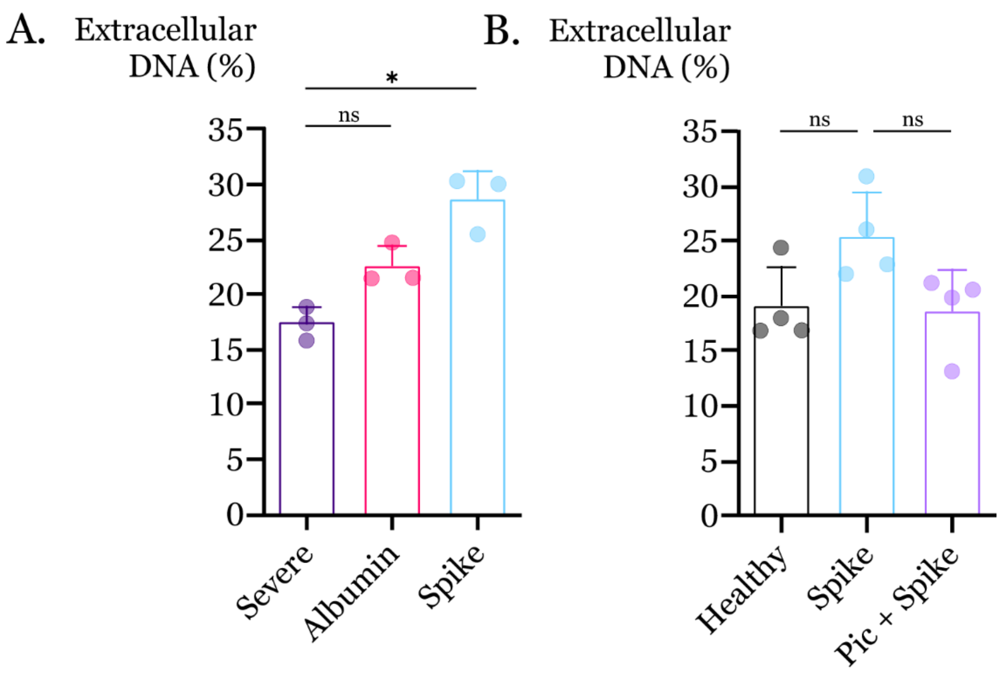

Figure S8

Control experiments to Figure 6F. Neutrophils isolated from healthy donors treated with (A) COVID-19 plasma and spike protein (100 µg/ml) or albumin (100 µg/ml) or (B) plasma from healthy controls and spike (100 µg/ml) alone or together with piceatannol (Pic) (10 µM) for 5 hours. Induced NET formation was determined by released extracellular DNA quantified as percentage of positive control. Mean value of (A) neutrophil donors (n = 3) each treated with plasma from 3 patients with severe COVID-19 or (B) neutrophil donors (n = 4) each treated with plasma from 4 healthy controls. (B) Statistical significance was determined by Repeated measures one-way ANOVA (Friedman test) with Dunn's multiple comparisons test (*$p \leq 0.05$).

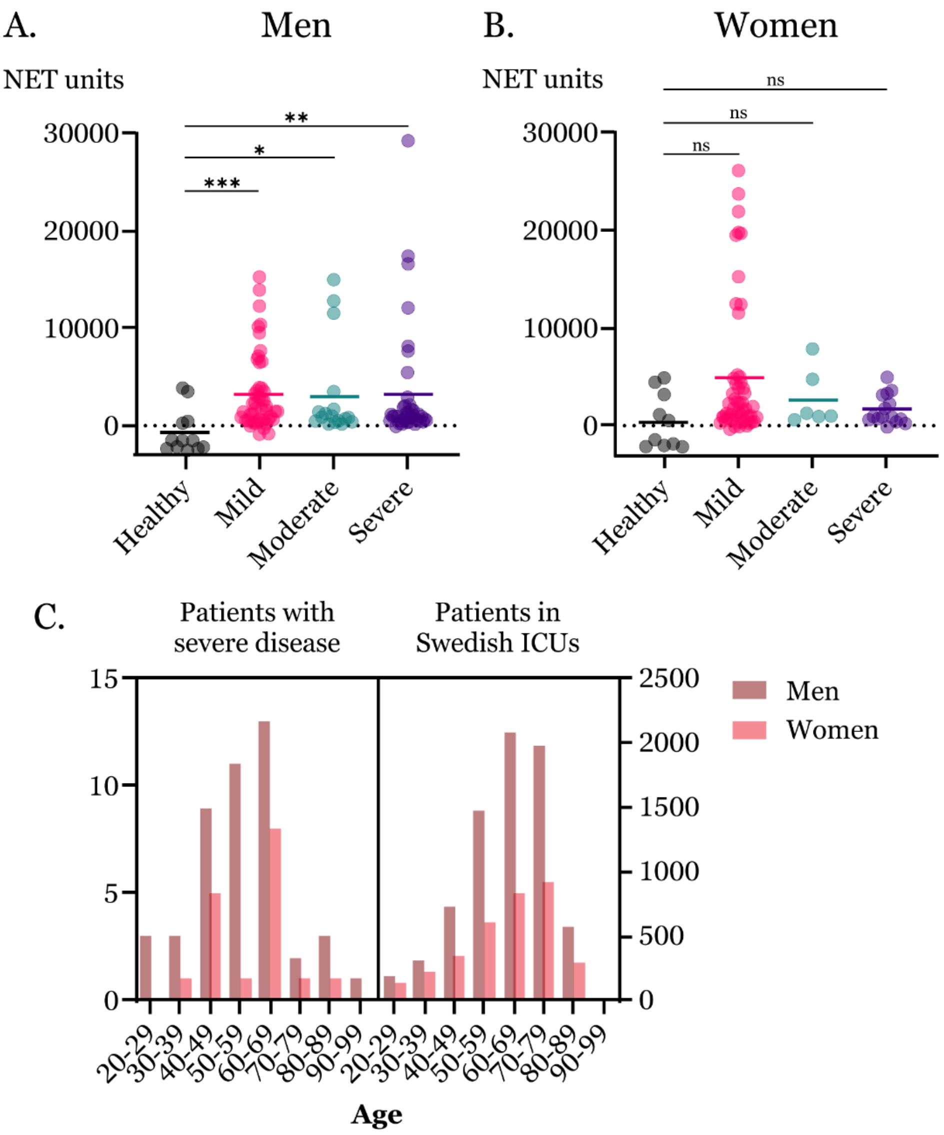

Figure S9

(A, B) MDCs in plasma of COVID-19 patients and healthy controls quantified by sandwich ELISA. Maximum value within 30 days after disease onset in plasma from (A) men with mild (n = 49), moderate (n = 17) or severe (n = 41) disease and healthy controls (n = 12) and (B) women with mild (n = 52), moderate (n = 6) or severe (n = 15) disease and healthy controls (n = 10). Statistical significance was determined by Kruskal-Wallis test with Dunn's multiple comparisons test (*$p \leq 0.05$, **$p \leq 0.01$, ***$p \leq 0.001$).
(C) Age and sex distribution of patients with severe COVID-19 analyzed for MDCs (left) and in Swedish ICUs (right) between january 2020 and march 2024.

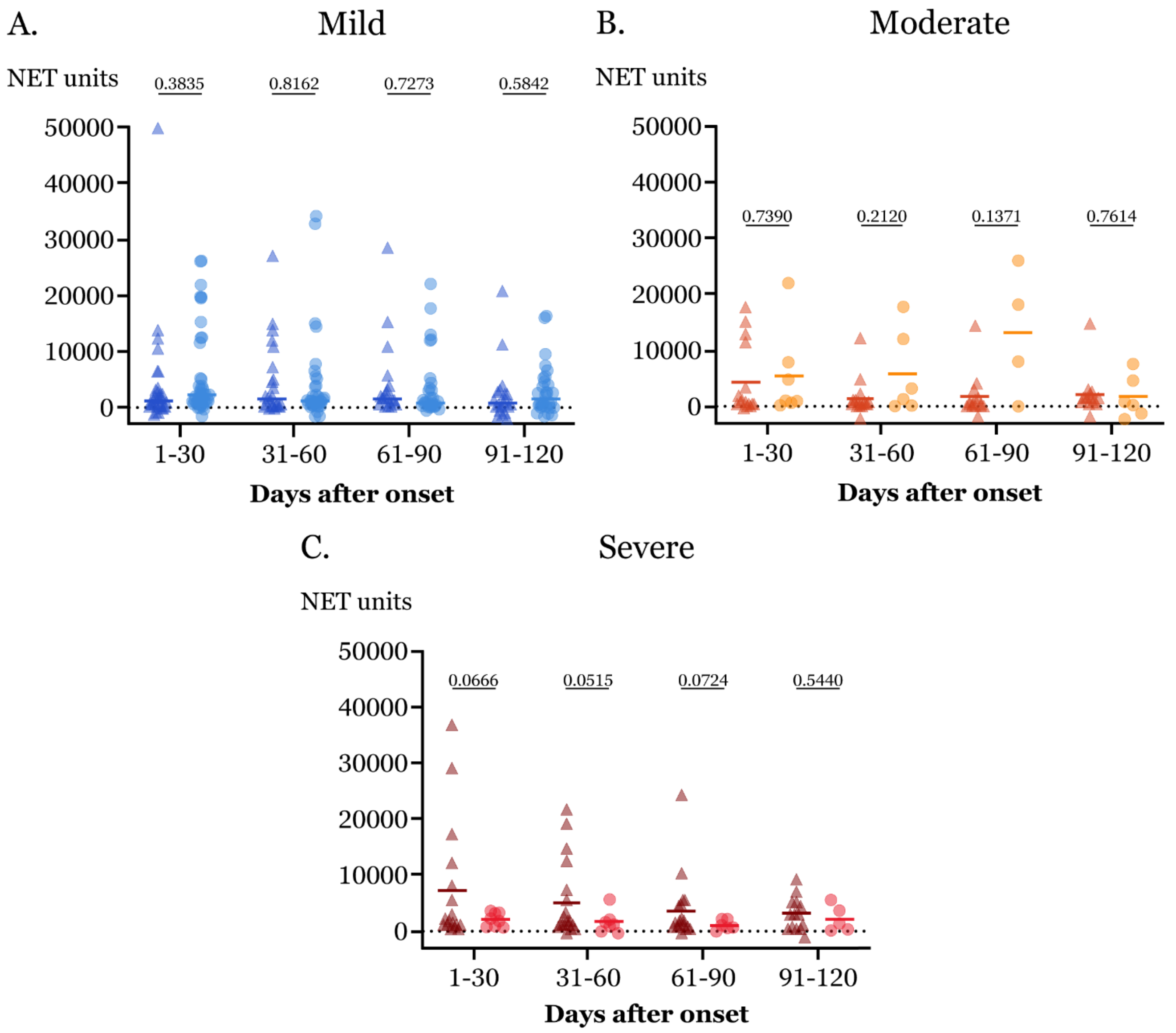

Figure S10

MDCs in plasma of COVID-19 patients quantified by sandwich ELISA. Patients with (A) mild, (B) moderate and (C) severe disease evaluated for differences between sexes in cosecutive samples within 120 days after onset while triangles depict samples of men and circles depict samples of women. Maximum value per patient for each time period (patients sampled only once were excluded) where statistical significance was determined by Mixed-effects model with Tukey's multiple comparisons test (*p ≤ 0.05).

Table S1. Baseline characteristics of the study cohort, divided by sex.

	**Overall** n = 206*	**Men** n = 120*	**Women** n = 86*	**P value**×
Age	47 (33, 60)	48 (36, 62)	45 (32, 59)	0.136
BMI[1]	27 (24, 31)	28 (25, 31)	25 (23, 30)	**0.021**
Diabetes	14 (6.8%)	8 (6.7%)	6 (7.0%)	0.931
Hypertension	50 (24%)	31 (26%)	19 (22%)	0.537
Cardiovascular disease[2]	20 (9.7%)	16 (13%)	4 (4.7%)	**0.038**
Chronic lung disorder[3]	38 (18%)	20 (17%)	18 (21%)	0.437
Asthma	36 (17%)	20 (17%)	16 (19%)	0.718
Autoimmune disease[4]	10 (4.9%)	4 (3.3%)	6 (7.0%)	0.326
Immunocompromised[5]	1 (0.5%)	0 (0%)	1 (1.2%)	0.417
Malignancy[6]	3 (1.5%)	1 (0.8%)	2 (2.3%)	0.572
Charlsons score				0.310
0	157 (76%)	90 (75%)	67 (78%)	
1-2 mild	45 (22%)	29 (24%)	16 (19%)	
3- moderate/severe	4 (1.9%)	1 (0.8%)	3 (3.5%)	
Smoking status[7]				**0.003**
Current smoker	4 (2.1%)	1 (0.9%)	3 (3.8%)	
Former smoker	49 (25%)	38 (33%)	11 (14%)	
Non-smoker	141 (73%)	77 (66%)	64 (82%)	
Level of education[8]				**0.005**
Lower	14 (7.6%)	8 (7.3%)	6 (7.9%)	
Medium	84 (45%)	60 (55%)	24 (32%)	
Higher	87 (47%)	41 (38%)	46 (61%)	
Highest infection severity				**0.002**
Healthy controls	7 (3.4%)	5 (5.8%)	2 (1.7%)	
Mild	108 (52%)	56 (65%)	52 (43%)	
Moderate	29 (14%)	8 (9.3%)	21 (18%)	
Severe	62 (30%)	17 (20%)	45 (38%)	
Hospitalized	91 (44%)	66 (55%)	25 (29%)	**<0.001**
Leukocyte count[9]	6.00 (4.98, 7.93)	6.20 (5.14, 8.96)	5.62 (4.55, 7.14)	**0.030**
Neutrophil count[9]	3.70 (2.78, 5.93)	3.90 (3.00, 6.47)	3.25 (2.49, 4.56)	**0.008**

[*] Median (IQR); n (%), n, number of patients; IQR, interquartile range[1] Body Mass Index is missing in 2 patients. The analysis is based on 204 patients.
[2] Ischemic heart disease, congestive heart failure, arrythmias, aortic disease, valvular heart disease or peripheral arterial insufficiency.
[3] Chronic obstructive pulmonary disease and asthma.
[4] Including rheumatic diseases.
[5] Immune deficiency diseases or immunosuppressive/immunomodulatory medication.
[6] Solid localized tumor, lymphoma, or leukemia
[7] Smoking status is missing in 12 patients. The analysis is based on 194 patients.
[8] Level of education is missing in 21 patients. The analysis is based on 185 patients. Lower: Less than three years beyond Swedish compulsory school. Medium: Three years beyond Swedish compulsory school, but no college or university degree. Higher: University or college degree.
[9] Missing in 39 patients. The analysis is based on 167 patients. Mean value of leukocyte and neutrophil counts in samples collected during the first 30 days after infection onset.
[×] Wilcoxon rank sum test; Pearson's Chi-squared test; Fisher's exact test.

Table S2. Infection severity and respiratory support in patients with severe COVID-19, divided by sex.

	**Overall** n = 62[1]	**Men** n = 45[1]	**Women** n = 17[1]	**P value**[2]
Extracorporeal Membrane Oxygenation	1 (1.6%)	0 (0%)	1 (5.9%)	0.274
Mechanical ventilation	12 (19%)	9 (20%)	3 (18%)	>0.999
Days in mechanical ventilation	15 (6, 21)	15 (9, 22)	6 (3, 14)	0.308
Non-invasive ventilation	6 (9.7%)	5 (11%)	1 (5.9%)	>0.999
High-flow nasal cannula	61 (98%)	45 (100%)	16 (94%)	0.274
Length of hospital stay - days	9 (6, 16)	10 (6, 17)	9 (8, 12)	0.630

[1] n (%); Median (IQR), n, number of patients; IQR, interquartile range
[2] Fisher's exact test; Wilcoxon rank sum test

Table S3. Baseline characteristics of the study cohort, divided by initial infection severity.

Patients were classified as having mild, moderate, or severe COVID-19 according to WHO's clinical progression scale [1].

	**Overall** n = 206*	**Healthy controls** n = 7*	**Mild** n = 108*	**Moderate** n = 29*	**Severe** n = 62*	**P value**×
Age	47 (33, 60)	45 (35, 49)	38 (25, 51)	59 (47, 71)	59 (45, 65)	**<0.001**
Sex						**0.002**
Women	86 (42%)	5 (71%)	56 (52%)	8 (28%)	17 (27%)	
Men	120 (58%)	2 (29%)	52 (48%)	21 (72%)	45 (73%)	
BMI[1]	27 (24, 31)	24 (21, 28)	24 (22, 27)	30 (28, 32)	31 (28, 34)	**<0.001**
Diabetes	14 (6.8%)	0 (0%)	3 (2.8%)	4 (14%)	7 (11%)	**0.044**
Hypertension	50 (24%)	0 (0%)	14 (13%)	14 (48%)	22 (35%)	**<0.001**
Cardiovascular disease[2]	20 (9.7%)	0 (0%)	5 (4.6%)	5 (17%)	10 (16%)	**0.027**
Chronic lung disorder[3]	38 (18%)	1 (14%)	15 (14%)	8 (28%)	14 (23%)	0.231
Asthma	36 (17%)	1 (14%)	14 (13%)	8 (28%)	13 (21%)	0.202
Autoimmune disease[4]	10 (4.9%)	0 (0%)	4 (3.7%)	3 (10%)	3 (4.8%)	0.520
Immunocompromised[5]	1 (0.5%)	0 (0%)	0 (0%)	1 (3.4%)	0 (0%)	0.175
Malignancy[6]	3 (1.5%)	0 (0%)	1 (0.9%)	0 (0%)	2 (3.2%)	0.751
Charlsons score						**0.022**
0	157 (76%)	6 (86%)	91 (84%)	18 (62%)	42 (68%)	
1-2 mild	45 (22%)	1 (14%)	17 (16%)	10 (34%)	17 (27%)	
3-moderate/severe	4 (1.9%)	0 (0%)	0 (0%)	1 (3.4%)	3 (4.8%)	
Smoking status[7]						**<0.001**
Current smoker	4 (2.1%)	1 (14%)	2 (2.0%)	0 (0%)	1 (1.7%)	
Former smoker	49 (25%)	0 (0%)	12 (12%)	12 (44%)	25 (42%)	
Non-smoker	141 (73%)	6 (86%)	87 (86%)	15 (56%)	33 (56%)	
Level of education[8]						0.744
Lower	14 (7.6%)	0 (0%)	5 (5.2%)	2 (7.7%)	7 (13%)	
Medium	84 (45%)	2 (33%)	46 (47%)	12 (46%)	24 (43%)	
Higher	87 (47%)	4 (67%)	46 (47%)	12 (46%)	25 (45%)	
Hospitalized	91 (44%)	0 (0%)	0 (0%)	29 (100%)	62 (100%)	**<0.001**
Leukocyte count[9]	6.00 (4.98, 7.93)	NA (NA, NA)	5.26 (4.53, 6.25)	6.17 (4.75, 8.80)	8.47 (6.85, 11.09)	**<0.001**
Neutrophil count[9]	3.70 (2.78, 5.93)	NA (NA, NA)	3.00 (2.29, 3.51)	4.17 (3.39, 6.39)	6.49 (4.64, 7.93)	**<0.001**

* Median (IQR); n (%), n, number of patients; IQR, interquartile range

[1] Body Mass Index is missing in 2 patients. The analysis is based on 204 patients.

[2] Ischemic heart disease, congestive heart failure, arrythmias, aortic disease, valvular heart disease or peripheral arterial insufficiency.
[3] Chronic obstructive pulmonary disease and asthma.
[4] Including rheumatic diseases.
[5] Immune deficiency diseases or immunosuppressive/immunomodulatory medication.
[6] Solid localized tumor, lymphoma, or leukemia.
[7] Smoking status is missing in 12 patients. The analysis is based on 194 patients.
[8] Level of education is missing in 22 patients. The analysis is based on 184 patients. Lower: Less than three years beyond Swedish compulsory school. Medium: Three years beyond Swedish compulsory school, but no college or university degree. Higher: University or college degree.
[9] Missing in 39 patients. The analysis is based on 167 patients. Mean value of leukocyte and neutrophil counts in samples collected during the first 30 days after infection onset.
[×] Kruskal-Wallis rank sum test; Fisher's exact test.

Table S4. Values for mean rank diff. and adjusted P value for cytokines and NET components from Figure S7.

	Severity		Sex	
	Mean rank diff.	Adjusted P value	Mean rank diff.	Adjusted P value
**CCL8**	-17.57	0.06	12.35	0.56
**CXCL12**	-13.60	0.30	4.30	>0.99
**OLR1**	2.68	0.96	1.02	>0.99
**IL-27**	-16.39	0.10	7.89	0.98
**CXCL9**	-20.21	0.01	4.80	>0.99
**TGF-α**	-7.34	0.87	-0.61	>0.99
**IL-1β**	-8.94	0.76	4.74	>0.99
**IL-6**	-30.30	<0.01	16.21	0.10
**TNFSF12**	13.32	0.32	5.57	>0.99
**TSLP**	-5.50	0.89	-13.38	0.12
**CCL11**	11.45	0.47	0.84	>0.99
**HGF**	-32.04	<0.01	12.62	0.52
**FLT3LG**	26.06	<0.01	-7.35	0.98
**IL-7**	-23.48	<0.01	10.03	0.87
**IL-18**	-22.23	<0.01	14.99	0.19
**CCL13**	-0.87	0.96	-6.94	0.99
**TNFSF10**	23.55	<0.01	0.16	>0.99
**CXCL10**	-28.63	<0.01	12.12	0.58
**IFNG**	-19.03	0.02	8.21	0.97
**IL-10**	-22.78	<0.01	19.13	0.01
**CCL19**	-9.01	0.76	7.48	0.98
**TNF**	-13.26	0.32	5.44	>0.99
**IL-15**	-23.41	<0.01	12.17	0.58
**CCL3**	-25.22	<0.01	6.34	>0.99
**CXCL8**	-22.65	<0.01	6.07	>0.99
**MMP12**	24.32	<0.01	-8.25	0.97
**CSF2**	-12.42	0.37	-5.98	>0.99
**CSF3**	-22.09	<0.01	4.34	>0.99
**VEGFA**	-23.00	<0.01	10.03	0.87
**IL-17C**	4.77	0.94	4.93	>0.99
**EGF**	1.57	0.96	-0.25	>0.99
**CCL2**	-15.48	0.14	2.84	>0.99
**IL-17A**	-4.77	0.94	1.21	>0.99
**OSM**	-23.21	<0.01	7.66	0.98
**CSF1**	-22.72	<0.01	10.57	0.81
**CCL4**	-12.98	0.32	-0.30	>0.99
**CXCL11**	-25.71	<0.01	8.80	0.95
**LT-α**	23.62	<0.01	-8.94	0.95
**CCL7**	-33.09	<0.01	15.58	0.14
**MMP1**	-16.11	0.11	4.03	>0.99
**NET Units**	5.79	0.91	-12.01	0.58

Differences in cytokine and MPO-DNA complexes (MDCs) in plasma from COVID-19 patients with mild (n = 59) and severe (n = 19) disease or men (n = 45) and women first 30 days after disease onset. 40 cytokines quantified by Olink® and MDCs quantified by sandwich ELISA. Statistical significance was determined by Multiple Mann-Whitney tests with Holm-Šídák method.

References

1. Marshall, J.C., et al., *A minimal common outcome measure set for COVID-19 clinical research.* The Lancet Infectious Diseases, 2020. **20**(8): p. e192-e197.
2. Ermert, D., A. Zychlinsky, and C. Urban, *Fungal and Bacterial Killing by Neutrophils BT - Host-Pathogen Interactions: Methods and Protocols*, S. Rupp and K. Sohn, Editors. 2009, Humana Press: Totowa, NJ. p. 293-312.
3. Brinkmann, V., et al., *Neutrophil Extracellular Traps Kill Bacteria.* Science, 2004. **303**(5663): p. 1532-1535.
4. Rasband, W.S., *ImageJ*. 1997-2018, U. S. National Institutes of Health: Bethesda, Maryland, USA.
5. Wrapp, D., et al., *Cryo-EM structure of the 2019-nCoV spike in the prefusion conformation.* Science, 2020. **367**(6483): p. 1260-1263.
